# Systematic Review and Meta-Analysis on the Association between Outpatient Statins Use and Infectious Disease-Related Mortality

**DOI:** 10.1371/journal.pone.0051548

**Published:** 2012-12-17

**Authors:** Yu Ma, Xiaozhong Wen, Jing Peng, Yi Lu, Zhongmin Guo, Jiahai Lu

**Affiliations:** 1 Department of Epidemiology and Biostatistics, School of Public Health, Key Laboratory of Tropical Diseases Control Research, Ministry of Education, Sun Yat-sen University, Guangzhou, Guangdong Province, PR China; 2 Department of Population Medicine, Harvard Medical School and Harvard Pilgrim Health Care Institute, Boston, Massachusetts, United States of America; 3 Department of Epidemiology and Biostatistics, School of Public Health, Sun Yat-sen University, Guangzhou, Guangdong Province, PR China; 4 School of Public Health and Tropical Medicine, Southern Medical University, Guangzhou, Guangdong Province, PR China; 5 Laboratory Animal Center, Sun Yat-Sen University, Guangzhou, Guangdong Province, PR China; University of Hong Kong, Hong Kong

## Abstract

**Background:**

To update and refine systematic literature review on the association between outpatient statins use and mortality in patients with infectious disease.

**Materials and Methods:**

We searched articles published before September 31, 2012, on the association between statins and infectious disease-related mortality through electronic databases. Eligible articles were analyzed in Review Manager 5.1. We conducted stratification analysis by study design, infection types, clinical outcomes and study locations.

**Results:**

The pooled odds ratio (OR) for death (statins use vs. no use) across the 41 included studies was 0.71 (95% confidence interval: 0.64, 0.78). The corresponding pooled ORs were 0.58 (0.38, 0.90), 0.66 (0.57, 0.75), 0.71 (0.57, 0.89) and 0.83 (0.67, 1.04) for the case-control study, retrospective cohort studies, prospective cohort studies and RCTs; 0.40 (0.20, 0.78), 0.61 (0.41, 0.90), 0.69 (0.62, 0.78) and 0.86 (0.68, 1.09) for bacteremia, sepsis, pneumonia and other infections; 0.62 (0.534, 0.72), 0.68 (0.53, 0.89), 0.71 (0.61, 0.83) and 0.86 (0.70, 1.07) for 30-day, 90-day, in-hospital and long-term (>1 year) mortality, respectively.

**Conclusions:**

Outpatient statins use is associated with a lower risk of death in patients with infectious disease in observational studies, but in a less extent in clinical trials. This association also varies considerably by infection types and clinical outcomes.

## Introduction

Severe infections always remain a major cause of morbidity, mortality, and economic burden worldwide. For example, there are nearly 751,000 cases of sepsis in the United States each year and the number of cases is still increasing by 1.5% per year, resulting in an annual estimated cost of $16.7 billions [1]. Despite new advances in antimicrobial therapy and medical management, only 50%–70% patients can survive from sepsis [2]. In addition, Pneumonia induced by influenza and chronic obstructive pulmonary disease (COPD) had caused thousands of deaths in several epidemics [3], such as 1918 influenza epidemic, Asia influenza during 1957–1958, Hong Kong influenza in 1968, and Spanish influenza in 1942.

Statins, as one of the inhibitors of 3-hydroxy-3-methylglutaryl coenzyme A reductase (HMG-CoA), are traditionally used to lower the level of blood cholesterol in patients with cardiovascular diseases or to prevent cardiovascular events. Recently, statins have been proposed as novel therapeutic and preventive agents for infection, given the increasing evidence, mostly from observational studies, that statins are associated with a lower mortality in patients with infectious disease [4]–[7]. Researchers believe that statins can mitigate the inflammatory response in patients with sepsis or COPD, which reflects its anti-inflammatory and immunoregulation effects [8]. This may occur through several possible biological mechanisms. Firstly, statins block the mevalonate pathway by inhibiting HMG-CoA reductase, which interfere with the recognition of microbial products by immune cell. They can also decrease production of proinflammatory cytokines such as tumor necrosis factor α (TNF-α), interleukin1 (IL-1), and IL-6 present during sepsis and COPD, and thus depress the inflammatory cascade [9]. Secondly, the antioxidant and anti-apoptotic properties of statins blunt the effects of sepsis [10]. Thirdly, the antithrombotic properties of statins decrease the effect of sepsis-induced coagulopathy [11]. Finally, statins increase the physiologic concentrations of nitric oxide (NO) by increasing the expression of endothelial NO synthase and down-regulating inducible NO synthase, and thus reverse the endothelial dysfunction in sepsis [12].These pleiotropic effects of statins have been demonstrated in experimental models (in vitro and in vivo), and some [13]–[15] but not all studies [4], [6], [7], [16], [17] showed serendipitous benefits of statins to patients with severe infections, such as sepsis and COPD. However, it is unclear which of them can explain the association between statins and lower infectious disease-related mortality observed in clinical observational studies.

**Figure 1:**
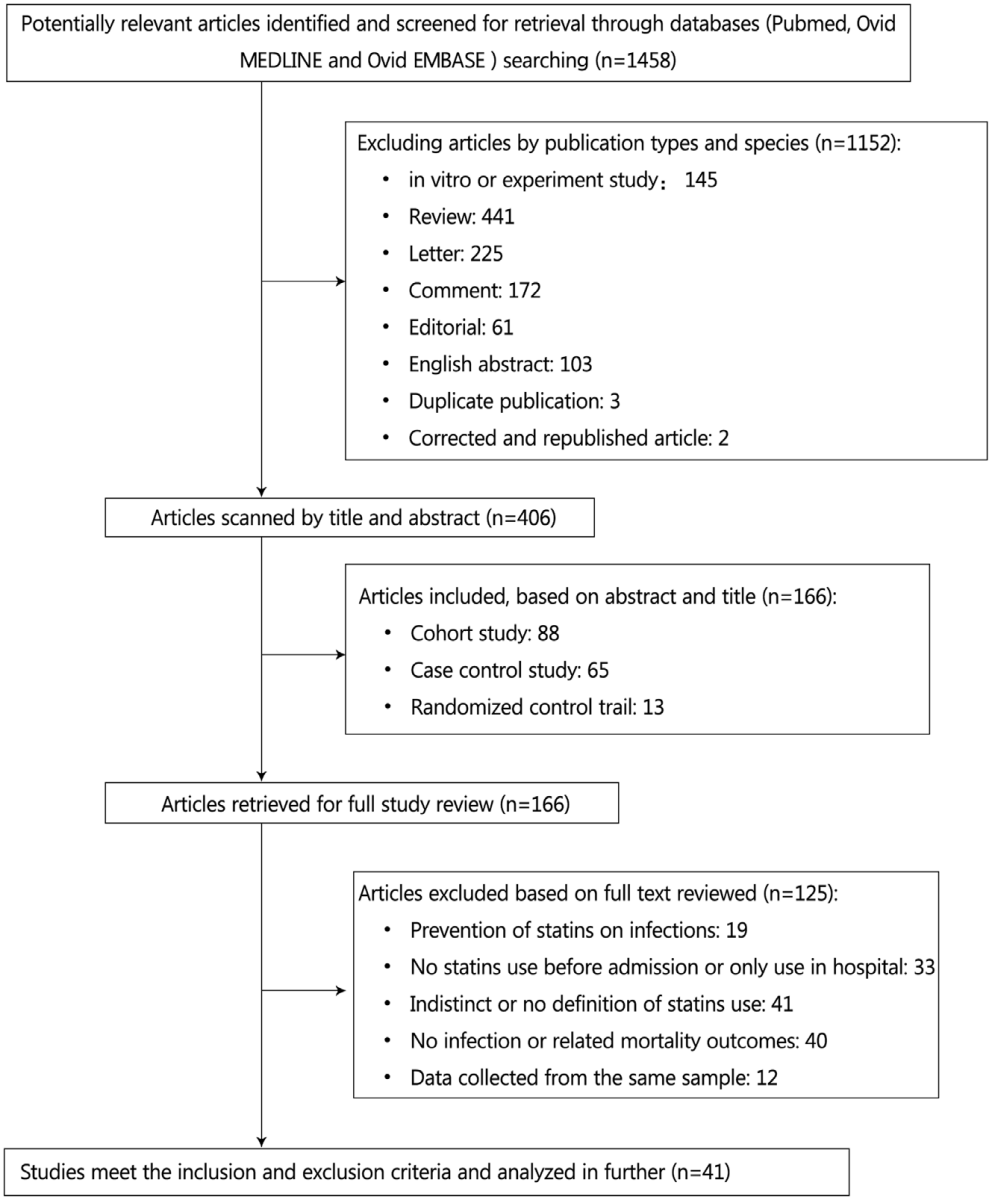
Flowchart of included studies and selection progress.

So far, there are 4 published systematic reviews (3 of them are quantitative analyses) on the studies on the association between statins and infectious disease-related mortality [18]–[21]. Tleyjeh et al. [18] reviewed 9 cohort studies published as of 2007 that examined the effect of statins on infection-related mortality by bacteremia (n = 3), pneumonia (n = 3), sepsis (n = 2), and bacterial infection (n = 1). The pooled effect estimate (odds ratio or hazard ratio of mortality) was 0.55 (95% CI, 0.36–0.83) in favor of statins. Kopterides et al. [19] reviewed 15 studies published as of 2008 that examined statins and infection-related mortality: 10 of them reported protective effects of statins, 4 showed null effects, and 1 showed risk/adverse effects. Janda et al. [20] reviewed 20 studies on severe infections and sepsis published as of 2009. Among them, 15 studiesreported protective effects of statins [4], [7], [13]–[15], [22]–[31]: 7 ones on 30-day mortality (OR, 0.61 [95% confidence interval or CI, 0.48–0.73]), 7 ones on in-hospital mortality (OR, 0.38 [95% CI, 0.13–0.64]). Bjorkhem et al. [21] reviewed 15 observational studies comprising 113 910 patients between 2001 and 2009, and found statin use was associated with a significantly (*P*<0.0001) reduced mortality in patients suffering from bacterial infections (OR, 0.52 [95% CI, 0.42–0.66]) but no longer significant after adjusting for publication bias by precision estimate test (OR, 0.79, [5% CI, 0.58–1.07]). However, these systematic reviews have not sufficiently addressed the 3 critical challenges in this field: potential time-varying effects of statins, the differential effects of statins by types of infections, conflicting by results in different study design. Answers to these 3 questions have important implications: they can help us to better interpret (e.g. causality) on the observed associations, and can also influence how physicians, patients, and public health policy makers use the existing evidence. Moreover, only 1 randomized clinical trial (RCT) was included in previous reviews. Given the high validity of RCTs, it is important to include more recently published RCTs into the review/meta-analysis to provide a more conclusive interpretation on the potential effects of statins on infectious disease-related mortality.

Therefore, this meta-analysis aimed 1) to update the literature as of September 2012; 2) to summarize the association between outpatient statins use and infectious disease-related mortality across all published studies (observational studies and RCTs); 3) to examine whether this association differ by study design, infection types, outcome measures and study locations.

## Methods

The systematic review and meta-analysis was performed according to the recently published recommendations and checklist of the Preferred Reporting Items for Systematic Reviews and Meta-Analyses (PRISMA) statement.

**Table 1:** Characteristics of 41 included studies.

Author	Type of infection	Study design	Country	Study settings	Sample size		Mean age	Sex ratio
(study year)					Statin user	Non-user		(male : female)
Martin [2](2003–2004)	sepsis	RC	U.S.A	University Hospital and Health System	16	37	59.5	1.52∶1
Frost [4](1992–2003)	Influenza and COPD	MRC^3^	U.S.A	Health data library (LPD and HMO)	19,058	57,174	Not mentioned	1.09∶1
Kruger [5](2000–2003)	bacteremia	RC^2^	Oceania	2 hospital	66^a^56^b^	372	Not mentioned	Not mentioned
Thomsen 2006 [6](1997–2004)	bacteremia	PC^1^	Denmark	population-based health registries	176	5,177	72	1.21∶1
Majumdar [7](2000–2002)	pneumonia	PC^1^	Canada	6 local hospital	325	3,090	75	1.12∶1
Mortensen 2007 [13](2000)	sepsis	RC^2^	U.S.A	Veterans Affairs health care networks	480	2471	74.4	69.19∶1
Mortensen 2008 [14](1999–2000)	pneumonia	RC^2^	North America	Health data library	3,728	4,924	75.2	1∶1.44
Liappis [15](1995–2000)	bacteremia	RC^2^	U.S.A	Veterans’ Affairs Medical Center in Washington, DC	35	353	63	193∶1
Myles [16](2001–2002)	pneumonia	RC^2^	Britain	Health data library	357	3,324	>40	Not mentioned
Yende [17](2001–2003)	CAP and sepsis	MC^4^	U.S.A	28 hospitals in 4 cities	426^a^354^b^	1,469^a^1,541^b^	67.25	1.08∶1
Chalmers [22](2005–2007)	pneumonia	PC^1^	Britain	NHS Lothian University Hospitals	257	750	66	1∶1.01
Dobesh [23](2005–2006)	sepsis	RC^2^	U.S.A	ICU in teaching hospita	60	128	66.5	1.14∶1
Donnino [24](2003–2004)	Emergency infection	PC^1^	U.S.A	Emergency department	474	1562	61	1∶1.07
Schlienger [25](1995–2002)	pneumonia	NCC^5^	Britain	based database (GPRD)	156	412	Not mentioned	1.18∶1
Hsu [26](1995–2006)	Organ transplantation infection	RC^2^	U.S.A	University of Wisconsin	80	231	51	187∶124
Mortensen 2005 [27](1999–2002)	pneumonia	RC^2^	U.S.A	2 academic tertiary care hospitals	110	677	60	3.74∶1
Almog [28](2001–2003)	infection	PC^1^	Israel	School medicine center	5,698	5,664	65	1.70∶1
Thomsen 2008 [29](1997–2004)	pneumonia	RC	Denmark	Medical record library	1,371	28,529	73	1∶2.39
Tseng [30](2004)	sepsis (subarachnoid hemorrhage)	Clinical trail	Britain	Addenbrooke’s Hospital	40	40	52.9	1∶1.22
Schmidt [36](2006)	MODSE infection	MRC^3^	Germany	ICU in teaching hospital	40	80	64.6	2.63∶1
Douglas [37](1995–2006)	pneumonia	RC^2^	Britain	Health data library	942	3,615	65	Not mentioned
Kwong [38](1996–2006)	Influenza	MRC^3^	Canada	4 health data library	1,120,319	1,120,319	74.34	821.57∶1
Fernandez [39](2002–2004)	ICU infection	RC^2^	Spain	Medical-surgical ICU	38	400	62.38	2.33∶1
Yang [40](2001–2002)	sepsis	RC	Taiwan	Hospital of Taiwan University	104	350	64.23	1.20∶1
Luc de Saint [41](2009)	acute infection	PC^1^	France	One tertiary health center	139	782	71.65	1.13∶1
Fellstrom [42](2005–2011)	multiple infection	RCT^6^	U.S.A	280 centers in 25 countries	1,389	1,384	64	1.64∶1
Kjekshus [43](2003–2007)	Infection	RCT	U.S.A	Several hospitals	2,514	2,497	73	3.25∶1
Hollis R. [44](2006–2008)	sepsis	PC^1^	U.S.A	From VALID study	149	426	59.07	1.28∶1
Sharon [45](2008–2009)	bloodstream infection	RC^2^	U.S.A	2 hospital	447	458	69.76	1.04∶1
GISSI [46](2002–2008)	moltiple infection	RCT	U.S.A	Fom GISSI-HF trial	2,285	2,289	68	3.43∶1
Wanner [47](2000–2004)	Infection from hemodialysis	RCT	Germany	178 centers	619	636	66	1.17∶1
Holdaas [48](1997–2003)	Infection	RCT	Northern Europe and Canada	84 centers	1,050	1,052	48	1.97∶1
Serruys [49](1996–1998)	Infection	RCT	Europe, Canada, Brazil	57 interventional centers in 10 countries	844	833	60	5.19∶1
Stegmayr [50](1998–2000)	Infection (severe renal failure)	RCT (not blind	Sweden	Not mentioned	70	73	68	2.33∶1
Michael B. [51](2003–2005)	Pneumonia	RC^2^	USA	376 acute care facilities in the US	23,285	97,969	74	1∶1.28
Nielsen [52](1997–2009)	Pneumonia	PC^1^	Denmark	Danish patient registration system and civil registration system	7,223^c^1,903^d^	61,827	Not mentioned	1.131∶1
Vandermeer [53](2007–2008)	Influenza	surveillance	USA	59 counties in 10 states	1013	2030	70.4	1∶1.27
Brett [54](2009–2010)	Influenza A	RCC^7^	UK	75 hospitals in 31 cities	477	94	52	1∶1.27
Sever [55](2000–2010)	Infection/respiratory illnes	RCT	UK	multicenter	2234	2198	Not mentioned	Not mentioned
Mortensen 2009 [56](1998–2000)	COPD exacerbation	RC^2^	USA	Veterans Affairs administrative data	4,711	6,501	74	49∶1
Makris [57](2008–2010)	ICU infection	RCT	Greece	2 centers	71	81	56	2.30∶1

**Type of study:** 1. perspective cohort study, 2. retrospective cohort study, 3. matched retrospective cohort study, 4. multicenter cohort study, 5. nested case control study, 6. randomized placebo controlled trials, 7. retrospective case-control study.

**Statins use:** a. prior use of statin, b. continued use of statin, c. current user d. former user.

### Search Strategy

All searches were limited to “English language” and “humans”. Two researhers conducted literature search independently in 4 steps. Step 1, we searched abstracts of published articles in English language through Pubmed, Ovid MEDLINE, and Ovid EMBASE databases, using the following key terms: (statin OR statins OR mevastatin OR simvastatin OR lovastatin OR fluvastatin OR rosuvastatin OR cerivastatin OR pravastatin OR atorvastatin OR hydroxymethylglutaryl-CoA Reductase Inhibitors) AND (infection OR sepsis OR bacteremia OR pneumonia OR virus infectious diseases OR respiratory tract infections OR organ transplantation OR). The final date of literature search was September 31 2012. Step 2, we excluded reviews, letter, comments, editorials, corrected publications, duplicate publications and abstracts only. Step 3, we screened all the remaining abstracts and selected those that met our eligibility criteria (see exclusion criteria below). Step 4, we searched the full-text articles of these eligible abstracts.

### Study Selection

Our included surveillance studies, cohort studies, case-control studies, and RCTs that focused on statins and infectious diseases. Infectious diseases in this Meta-analysis included phenomena, COPD, sepsis, bacteremia, respiratory infection, organ transplantation or any open surgery induced infection. Outpatient statins use was defined as use of statins before the admission or both before and during the hospitalization. We excluded studies that, 1) only focused on the preventive effects of statins on the incidence of infectious disease; 2) only had information on statins use during the hospitation for observational study; 3) did not provide clear definition of statins use; 4)had no outcome measures for infection-related mortality.

### Data Extraction and Quality Assessment

Two researchers (Ma Y and Peng J) independently reviewed the included studies and used the same Excel spreadsheet to extract relevant information of each study, including study objective, sample demographics (i.e. age, gender), comorbidities, type of infection, timing and dose of statins use, use of other medications or treatments, outcome measures (30-day, 90-day, in hospital and long-term mortality), association scale (OR or HR), and adjusted confounders. Disagreement between the 2 reviewers was first resolved by consensus. If consensus could not be reached, a senior reviewer (Guo Z) was consulted for the final decision. For observational studies, we used Newcastle-Ottawa Quality Assessment Scale (NOS) [32] to assess their quality. NOS has 8 questions reflecting 3 domains: quality of subject selection, comparability between two groups, and reliability of exposure or clinical outcomes. The full score of NOS is 9, and a score 8–9 is rated as excellent, 6–7 as good, 5 or below as fair. For clinical trials, we used Jadad Score [33] with 3 questions to assess the quality of randomization, blinding, and withdrawals or dropouts The full Jadad Score is 5, with 3–5 being rated as high quality and 1 or 2 as low quality.

**Table 2:** Definitions of exposure, outcomes, adjusted confounders, and quality score of 41 included studies.

Author	Exposure	Mortality outcome	Adjusted OR/HR (95% CI)	Adjust confounders	Quality score
Martin [2](2003–2004)	had been taking statins before admission	in-hospital	0.63 (0.19, 2.07)	1, 2, 5, 14, 9	8
Frost [4](1992–2003)	Individuals with at least 90 days of cumulative statin exposure prior to death or disenrollment [low daily dose (<4 mg/d) and moderate daily dose (>4 mg/d)]	influenza/pneumonia related	0.60 (0.34–1.06)0.73 (0.47–1.13)	2,6,5,13	9
Kruger [5](2000–2003)	statin was continued used during admission	bacteremia relatedin-hospital	0.29^e^ ‡ (0.10–0.86)0.09^e◊^ (0.01–0.64)0.39^f^ ‡ (0.17–0.91)0.06^f◊^ (0.01–0.44)	1,5,12	7
Thomsen 2006 [6](1997–2004)	current statin use as at least 1 filled prescription within 125 days before the hospitalization with pneumonia. Patients who filled at least 1 statin prescription more than 125 days before the hospitalization were classified as former statin users.	30-days	0.93 (0.66–1.30)	1,2,3,5,16	9
Majumdar [7](2000–2002)	taken for at least one week before admission to hospital and continued during hospital stay	in-hospital	1.10 (0.76–1.60)	1,2,3	8
Mortensen 2007 [13](2000)	received at least one active and filled prescription within 90 days of admission	30-days	0.48 (0.36–0.64)	1,2,3,5	8
Mortensen 2008 [14](1999–2000)	received at least one active and filled medicationwithin 90 days of admission	30-days	0.58 (0.42–0.80)	1,2,18,5,6,17	8
Liappis [15](1995–2000)	taking a statin at the time of admission and continued use of statin throughout the course of hospitalization	bacteremia related all cause	0.13 (0.02–0.99)	1,3,5,10,11,12	7
Myles [16]	Current exposure: when the most recent prescription was within 30 days before the pneumonia index dateRecent exposure: Prescriptions within 31–90 days before the index datePast exposure: any prescriptions dating more than 90 days before the index date	30-days	0.33^a^ *(0.19–0.58)0.58^a^ †(0.34–0.99)1.36^a♦^(0.86–2.16)	1,2,4,5,6	9
(2001–2002)		long-term	0.45^d^ *(0.32–0.62)0.62^d^ †(0.43–0.89)1.13^d♦^(0.77–1.65)		
Yende [17](2001–2003)	Prior use: a history of statin use in the week before admissionContinued use: continued use of statin after admit of hospital	90-days	0.90^b^ ‡(0.63–1.29)0.73^b◊^(0.47–1.13)	1,2,3,6,7,8,18,19	9
Chalmers [22](2005–2007)	Did not defined specifically	30-days	0.46 (0.25–0.85)	1,3,5,7	7
Dobesh [23](2005–2006)	received any statin or statin combination product at the time of admission or had been prescribed one of those products during hospitalization.	in-hospital	0.42 (0.21–0.84)	1,2,9	9
Donnino [24](2003–2004)	receive statin therapy during their inpatient hospital course	in-hospital	0.27 (0.1–0.72)	2,6,7	8
Schlienger [25](1995–2002)	received at least one prescription for a statin	fatal pneumonia related	0.47 (0.25–0.88)	3,4,5,15,	9
Hsu [26](1995–2006)	use of statins within 30 days prior to BSI	15-days	0.18 (0.04–0.78)	5,9,20	9
Mortensen 2005 [27](1999–2002)	had a statin listed on the electronic medical record (as an outpatient medication) or history and physical under outpatient medications.	30-days	0.36 (0.14–0.92)	3,4,7,16	8
Almog [28](2001–2003)	Individuals with at least 30 days of cumulative statin exposure prior to death or disenrollment	30-days infection-related	0.43 (0.13–1.38)	1,2,3,5,6,14	9
Thomsen 2008 [29](1997–2004)	Current use: use as at least 1 filled prescription within 125 days before the hospitalization with pneumoniaFormer use: filled at least 1 statin prescription more than 125 days before the hospitalization	30-days	0.69^a^ (0.58–0.82)	1,2,3,5,17,16	9
		90-days	0.75^b^ (0.65–0.86)		
Tseng [30](2004)	receive daily oral pravastatin (40 mg) or placebo for up to 14 days	6-month	0.12 (0.02, 0.69)		5
Schmidt [36](2006)	Did not defined specifically	28-days	0.53 (0.29–0.99)	not adjusted	7
Douglas [37](1995–2006)	received a prescription for a statin in the 60 day period before pneumonia to be users	within 6 months	0.67 (0.49–0.91)	1,2,3	9
Kwong [38](1996–2006)	received one or more prescriptions for a statin during the 90 days preceding the start of an influenza season	30-days	0.90^a^ (0.82–0.98)	1,2,3,5	9
		all-cause in influenza seasons	0.91^c^ (0.88–0.94)		
Fernandez [39](2002–2004)	taking statins before ICU admission and continuing on statin therapy throughout the course of hospitalization (40 mg per day)	ICU	2.30 (1.08–4.89)	5,9,12	9
Yang [40](2001–2002)	took a statin at least30 days before the sepsis episode and continued to receive statin therapy during the hospital course	30-days	0.95 (0.53–1.68)	1,2,3,5,11,12,14	9
Luc de Saint [41](2009)	patients under statin treatment at admission	in-hospital	0.98 (0.47–2.03)	1, 2, 12,	8
Fellstrom [42](2005–2011)	Rosuvastatin 10 mg v placebo	infection related	1.04^h^ (0.80–1.35)		5
Kjekshus [43](2003–2007)	Rosuvastatin 10 mg v placebo	infection related	0.79^h^ (0.55–1.12)		5
Hollis R. [44](2006–2008)	patients on any type of prehospital statin therapy were grouped as “statin users”	in-hospital	1.06^f^ (0.62–1.81)	1, 2, 4, 5, 9, 18	8
Sharon [45](2008–2009)	Administration of any statin medication at the time blood culture was sampling and/or documentation of statin use as an outpatient before hospitalization if the bacteremic blood culture was drawn within 24 hrs of admission	90-days	0.99^b^ (0.77–1.25)	1, 2, 3, 5, 15, 18	8
			0.86^g^ (0.66–1.12)		
GISSI [46](2002–2008)	Rosuvastatin 10 mg v placebo	infection related	1.50^h^ (0.77–2.95)		5
Wanner [47](2000–2004)	Atorvastatin 20 mg v placebo	infection related	0.91^h^ (0.65–1.26)		5
Holdaas [48](1997–2003)	Fluvastatin 40 mg v placebo	infection related	0.97^h^ (0.61–1.55)		4
Serruys [49](1996–1998)	Fluvastatin 80 mg v placebo	infection related	0.33^h^ (0.03–3.16)		5
Stegmayr [50](1998–2000)	Atorvastatin 10 mg v placebo	mortality of sepsis	0.57^h^ (0.18–1.95)		4
Michael B. [51](2003–2005)	at least one dose of any HMG-CoA reductase inhibitor on hospital day 1 or 2	In-hospital	0.86^f^ (0.79–0.93)	1, 2, 3, 4, 5, 7, 8	9
Nielsen [52](1997–2009)	Current use: at least one filled prescription with in 125 days of the pneumonia hospitalization/index dateFormer use: filled a prescription more than 125 days before hand	30 days	0.73* (0.67–0.79)0.91† (0.80–1.03)	1, 2, 3, 5, 6, 16, 17	9
Vandermeer [53](2007–2008)	Prior use: had a statin medication mentioned in their admission history and physicalContinued use: had any record of statin administration at any time during their hospitalization	Within 7 days	0.46 (0.23–.90)	1, 3, 13, 18, 21	8
		Within 14 days	0.51 (0.30–.88)		
		Within 21 days	0.60 (0.37–.97)		
		Within 30 days	0.59^a^ (0.38–.92)		
Brett [54](2009–2010)	Recorded in the case note current drug history	Influenza related	0.72 (0.38–1.33)	1, 2, 10, 15	8
Sever [55](2000–2010)	Atorvastatin 10 mg v placebo	Infection/respiratory	0.64^h^ (0.42–0.97)		5
		Infection	0.60^h^ (0.36–1.02)		
		respiratory	0.72^h^ (0.36–1.44)		
Mortensen 2009 [56](1998–2000)	Given medication if their last filled prescription included enough pills to last until the date of hospitalization	30-days	0.51^a^ (0.41–0.64)	1, 2, 5, 6, 17, 18	9
		90-days	0.51^b^ (0.40–0.64)		
Makris [57](2008–2010)	pravastatin sodium, 40 mg v placebo	30-days ICU treatment period	0.48^ah^ (0.21–1.09)		4

**Outcome:** a. 30-day mortality, b. 90-day mortality, c. all-cause mortality in influenza seasons, d. long-term mortality, e. Death attributable to Bacteremia, f. in-hospital mortality, g. mortality for sepsis, h. OR is calculated by events.

**Exposure:**

* current exposure

† recent or former exposure

♦ Past exposure

‡ prior use

◊ continued use.

**Adjusted confounders:** 1. Age, 2. Gender, 3. comorbid diseases, 4. smoking, 5. other drugs (antibiotics, aspirin, immunosuppressive agent, Angiotensin inhibitors, angiotensin-converting enzyme), 6. Charlson Comorbidity Index, 7. severity of disease, 8. other treatment in hospital, 9. APACHE score, 10. Crises signs, 11. time in ICU, 12. type of infection, 13. vaccine inoculation, 14. other library test data, 15. BMI, 16. alcohol drinking, 17. marital status, 18. race, 19. effect of health, 20. mental state, 21. antiviralinitiation.

### Statistical Analysis

We conducted quantitative data analyses in Review Manager 5.1 (StataCorp, College Station, Tex). We used *I^2^* statistic to test heterogeneity across the included studies. An *I^2^* value of 0% indicates absence of heterogeneity, while 25%, 50%, and 75% indicates low, moderate, and high heterogeneity, respectively [34]. Random effects model was used for high level of heterogeneity and fix effects model was used for low and moderate levels of heterogeneity. We estimated pooled odds ratio (OR) and the corresponding 95% confidence interval (CI) across studies by weighting the Odds ratios of each individual study according to their log-transformed inverse variance. We also conducted subgroup analyses by study design, infection types, outcome measures (30-day, 90-day, in-hospital mortality and long-term mortality), and study locations (grouped by continents). Whenever possible, we used the adjusted OR instead of the crude OR to calculate pooled ORs. We conducted sensitivity analysis with fail-safe number (Nfs) to calculate the number of negative studies needed to get opposite association between statins and mortality in patient with infectious disease. We also excluded single study or groups of studies based on the study design, outcomes, and quality score; re-ran the analysis to get new pooled ORs; and then compared the new pooled ORs with the original OR for all studies. This comparison can help to evaluate the appropriateness of inclusion and exclusion criteria as well as the stability of included studies. We assessed potential publication bias with Egger precision weighted linear regression tests and funnel plots [35].

## Results

We identified 1499 potential eligible articles at the initial search. Among them, there are 41 ones [2], [4]–[7], [13]–[17], [22]–[30], [36]–[57] met our inclusion and exclusion criteria (Figure 1), including 1 surveillance study, 2 case-control study, 19 respective cohort studies, 9 prospective cohort studies and 10 RCTs. Stratified by types of infection, 16 focused on pneumonia [4], [7], [14], [16], [17], [22], [25], [27], [29], [37], [38], [51]–[54], [56], 6 on bacteremia [5], [6], [15], [26], [36], [45], 7 on sepsis [2], [13], [23], [30], [40], [44], [50], and 12 on other infection [24], [28], [39], [41]–[43], [46]–[49], [55], [57]. Stratified by study locations, 20 studies were conducted in North America, 16 in Europe, 1 in Oceania, 2 in Asia, and 2 others cross continents. The average age of patients in these studies ranged from 40 to 75 years (Table 1).

**Figure 2:**
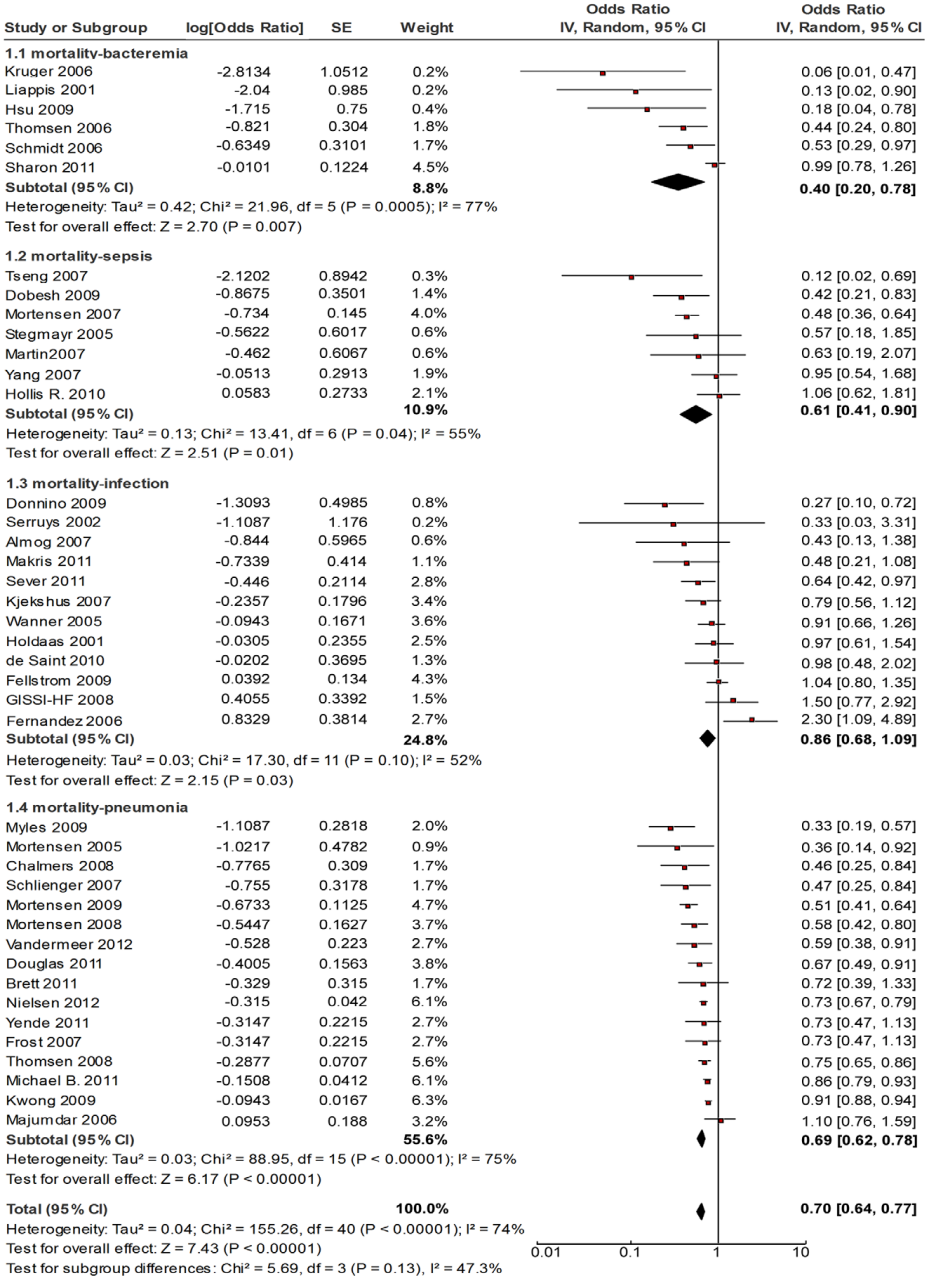
Forest plot of the association between statins and mortality for patients with infectious disease, by types of infection. Note: Each comparison was presented by the name of the first author and the year of the publication. The studies were shown by a point estimate of the OR and the accompanying 95% CI which were displayed on a logarithmic scale using a random effects model. The studies are sorted according to the estimate of OR. Between-study heterogeneity was tested by the *x^2^* -based Q-statistic, and its impact was quantified by *I^2^* which can range between 0 and 100%.

According to NOS, the 30 included observational studies were rated as excellent or good quality (score range, 7–9; mean, 8.15) (Table 2). According to Jadad scale, the 10 included clinical trials were rated as high quality (score range, 4–5; mean, 4.7), which represented good quality of these studies.

**Table 3:** Subgroup analyses by study design, types of infection, outcome measures and study location.

	n	Pooled OR	95% CI	Heterogeneity (*I^2^*)
**Study design**				
**Surveillance**	1	0.59	(0.38–1.33)	–
Retrospective cohort study	19	0.66	(0.57, 0.75)	82%
Prospective cohort study	9	0.71	(0.57, 0.89)	53%
Case-control study	2	0.58	(0. 38, 0.90)	0%
Clinical trial	10	0.83	(0.67, 1.04)	41%
**Type of infection**				
Bacteremia	6	0.40	(0.20, 0.78)	77%
Sepsis	7	0.61	(0.41, 0.90)	55%
Pneumonia	16	0.69	(0.62, 0.78)	75%
Other infection	12	0.86	(0.68, 1.09)	52%
**Outcome measure**				
30-day mortality	15	0.62	(0.54, 0.72)	77%
90-day mortality	5	0.68	(0.53, 0.89)	75%
In-hospital mortality	18	0.71	(0.61, 0.83)	78%
Long-term mortality	9	0.86	(0.70, 1.07)	39%
**Study location**				
North America	20	0.74	(0.65, 0.84)	78%
Europe	16	0.66	(0.57, 0.77)	55%
Australia	1	0.06	(0.01, 0.47)	–
Asia	2	0.76	(0.38, 1.53)	30%
Cross continents	2	0.93	(0.59, 1.46)	0%

The OR for death (statins use vs. no use) reported in the included studies ranged from 0.06 to 1.50 (Table 2). Twenty three studies found a protective effect of statins against death from infection, but the other 18 studies found null effects. There was substantial heterogeneity across the 41 studies (*I^2^* value, 74%), which supported the use of random effect model for meta-analysis. The overall pooled OR was 0.71 (95% CI: 0.64, 0.78) (Figure 2). Table 3 shows the subgroup-specific pooled ORs and their 95% CIs. Stratified by study design, the 10 RCTs showed null effect of statins (pooled OR, 0.83 [0.67, 1.04]), whereas the observational studies found beneficial effects: the subgroup-specific pooled ORs were 0.58 (0.38, 0.90) for the case-control study, 0.66 (0.57, 0.75) for retrospective cohort studies, and 0.71 (0.57, 0.89) for prospective cohort studies, respectively (Table 3). Stratified by types of infection, the protective effect of statins in bacteremia patients was stronger than other types of infections: the subgroup-specific pooled ORs were 0.40 (0.20, 0.78) for bacteremia, 0.61 (0.41, 0.90) for sepsis, 0.69 (0.62, 0.78) for pneumonia, and 0.86 (0.68, 1.09) for other infections, respectively (Figure 2). Stratified by types of outcome, the subgroup-specific pooled ORs were 0.62 (0.54, 0.72) for 30-day mortality, 0.68 (0.53, 0.89) for 90-day mortality, 0.71(0.61, 0.83)for in-hospital mortality and 0.86 (0.70, 1.07) for long-term mortality, respectively (Figure S1, S2, S3, S4, S5). Stratified by the quality of individual study, the pooled OR were 0.67 (0.59, 0.75) for observational studies rated as 8–9 score and 0.36 (0.18, 0.70) for studies rated as 7 score. Stratified by study locations, the pooled OR were 0.74 (0.65, 0.84) for studies in North America, 0.66 (0.57, 0.77) for studies in Europe, 0.06 (95%CI: 0.01, 0.47) for the study in Oceania, and 0.76 (95%CI: 0.38, 1.53) for studies in Asia, 0.93 (0.59, 1.46) for studies cross continents (Table 3).

**Figure 3:**
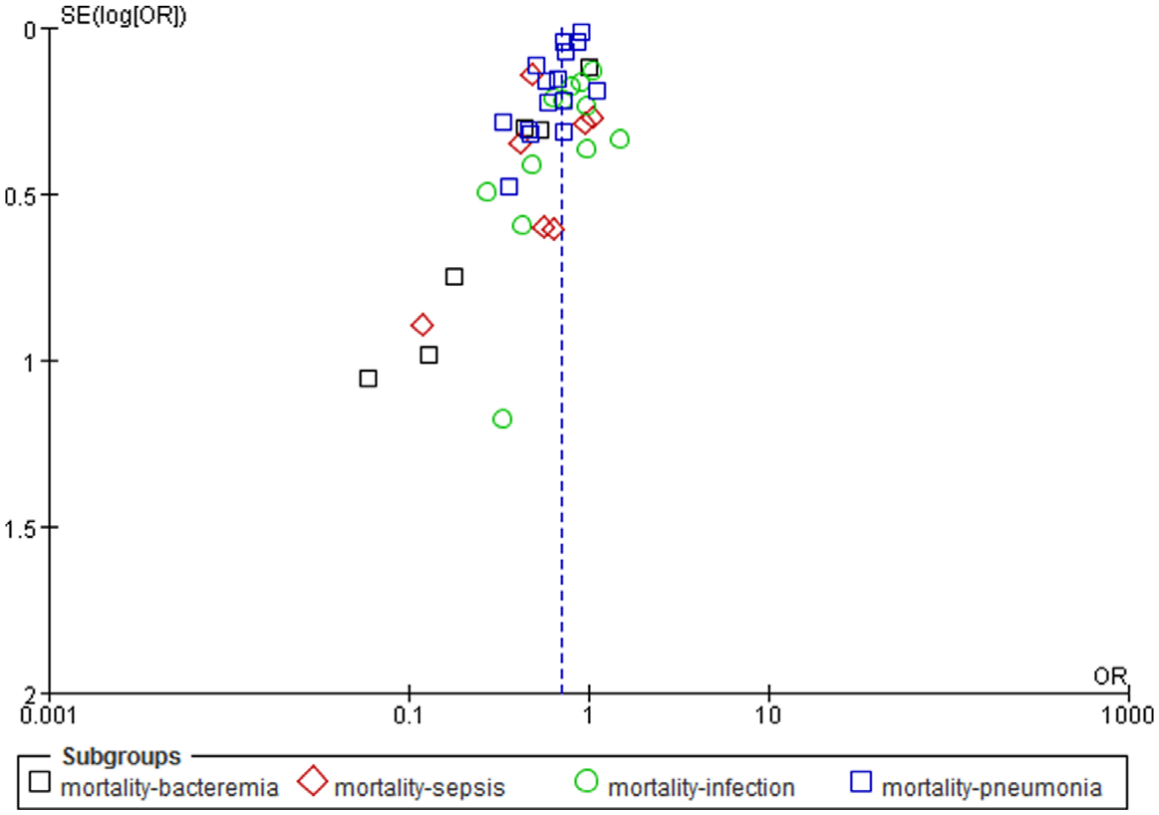
Funnel plot of the association between statins and mortality for patients with infectious disease, by types of infection.

There was substantial degree of heterogeneity across the included 41 studies (*I^2^* statistic, 74%). The *I^2^* statistic did not change considerably in the subgroup analyses based on total quality score, or subcategories of the quality score, i.e. exposure definition, outcomes, and confounding assessment. But the *I^2^* statistic was as low as 41% and 55%for clinical trials and studies in Europe, respectively. In our sensitivity analysis, there was no significant change in pooled OR when excluding any of the studies (data not shown) or any group of studies by infection type. Another sensitivity analysis showed that 938 negative studies were needed for getting the opposite association between statins and mortality in patient with infectious disease (Nfs = 938) and tolerance level was 103. Our Egger precision weighted linear regression tests showed the existence of publication bias (*P*-value <0.0001). Funnel plot also showed the absence of small studies in which statins might increase infectious disease-related mortality (Figure 3).

## Discussion

In this meta-analysis, we systematically reviewed 41 studies published during 2001–2012 on the association between statins use and infectious disease-related mortality. Overall, most observational studies found that statins were associated with lower mortality from infectious disease. Our pooled OR among these observational studies was similar to those in 3 previous meta-analyses by Tleyheh et al. [18], Surinder Janda et al. [20] and Bjorkhem et al [21]. However, we did not find conclusive evidence on this beneficial effect in clinical trials. In subgroup analysis, statins use was associated with lower 30-day, 90-day, and in-hospital mortality, but not with long-term mortality. Statins use was associated with lower mortality from bacteremia, pneumonia, and sepsis, but not with mortality from other infections and intensive care unit (ICU) patients.

We found that the magnitude of the association between statins use and infectious disease-related mortality tended to decrease with time, i.e. strongest for 30-day mortality followed by 90-day mortality and in-hospital mortality, and null for long-term mortality. This time trend suggests that the beneficial effect (if exists) of statins to lower infectious-disease related mortality may be short-term only. However, this time trend should be interpreted with caution due to potential misclassification of death outcome based on medical records, especially for in-hospital mortality. The substantial variation in hospitalization length posed a big challenge on estimating and interpreting pooled effect size of statins use, which can be confirmed by heterogeneity test within hospitalization period subgroup (*I^2^* is 78%, *P*<0.0001).

We also found the magnitude of the association between statins use and mortality was strongest in patients with bacteremia. This suggests that the protective effect of statins may be superior for bacteremia than other types of infection. Alternatively, this may be partially explained by less use of antibiotics in patients with bacteremia: only 1 out of 6 studies on bacteremia patients, reported antibiotics use before the admission to hospital or during hospital, compared with most studies among patients with other types of infections (such as 100% in Kwong study and 20% in Yende study).

In this analysis, we found the reported effects of statins use varied strikingly by study design. Observational studies consistently supported the beneficial effect of statins in lowering infection disease mortality, while all RCTs showed null effects. Though RCTs often provide more valid results, these 10 RCTs can not completely outweigh the evidence on the beneficial effect of statins from observational studies. These “null-effect” RCTs are often criticized for small sample size [58]. These criticisms usually are based on two factors. Firstly, the formal sample size calculations that compute the numbers of patients required prospectively, as if the trial had not yet been carried out. Secondly, the true power calculated when trial is over. Based on alpha level, sample size and actual rates of primary events among control and experimental patients, we did post-hoc analysis by re-calculating the power of 8 negative trials in 10 RCTs and found that only one trial’s retrospective power is bigger than 80% (Serrugs 81.5%), and all other 7 ones had power smaller than 80% (Fellstrom 8.9%, Kjekshus 25.5%, Wanner 14.3%, Stegmayr 47.3%, Holdaas 70.9%, Makris 60.5%). This under-power might be caused by the over-estimated event rate in control group and/or risk reduction level. In these 7 trials, investigator assumed the statins may reduce the risk of primary outcome by 20% to 50%, but the truth is the observed related risk reduction is obviously lower than 20% except for Makris study. This result indicated that the evidence to make a negative conclusion of statins is not sufficient and suggested retrospective sample size calculation is needed so as to add more representative patients in the trial. The sample size recalculation also showed that we still need 3–10 times of current number in each RCT to get positive result. Beside that, almost all these RCTs enrolled the patients who had existing cardiovascular disease. This could lead to misclassification of infection-related death, because of the difference between the primary and secondary infection. In addition, infection-related mortality is not a primary outcome in these studies on cardiovascular outcomes, and thus might not be measured accurately or appropriately.

Unexpectedly, we did not find significantly protective effect of statins against infection-related death in the 9 studies that focused on severe patient or patients admitted to ICU [7], [23], [26], [28], [36], [39], [40], [51], [57] respectively in bacterenia (0.38, 0.15–1.01), in sepsis (0.65, 0.29–1.44), in pneumonia (0.95, 0.84–1.07), but not in other infections subgroup (0.64, 0.44–0.92). One explanation can be that those severe patients usually have many severe complications [26], [39]. These complications often lead to adverse outcome and significantly increase the risk of death, which may dim the moderate protective effects of statins. Most interested us is that the result from only one clinical trial in these 9 studies provided evidence supporting that statins might affect the course of critically ill patients and decrees the ICU mortality [57]. Considering the differences in study design and implement between the observational studies and clinical trials, the most probable reason for this opposite result is the opportune moment of using statins. Patients in the observational studies might have already been using statins to treat high cholesterol (prevalent users). In clinical trials, however, statins were randomized to two groups after patients being recruited (new users). Another reason is the time difference between the progresses of critically ill and the onset time of statins. The immunomodulatory effects of statins can occur within 24 hours and thus acute treatment may down-regulate the level of pro-inflammatory cytokines [59]. That is why the acute curative effect in clinical trial is better than long-term effect in observational studies. These findings suggest that long-term use of statins may not be able to protect severe ICU patients against death from infection as common or less severe patients. However, we need more evidence from RCTs to confirm the acute effect of statin in reducing the ICU mortality among ICU patient.

The moderate heterogeneity across the included 41 studies may come from 2 main sources: study population and methodology. The significantly lower *I^2^* statistics in Europe studies than that in studies from other continents revealed the substantial difference in study population (e.g. ethnics and study locations), especially for limited source from Asia and Oceania population. For methodology, the low *I^2^* statistics in clinical trails indicated better quality control and more reliable results than observational studies. Other methodological heterogeneity included type of patients, type of infection, and dose of statins use across different studies.

### Study Strengths

This meta-analysis had several strengths. First, we included much more RCTs than previous reviews (8 vs. 0 in Tleyjeh et al.’s, 0 in Kopterides’, 0 in Bjorkhem and 1 in Janda et al.’s). Inclusion of these RCTs can substantially improve the validity of our analysis. Secondly, we conducted subgroup analyses by 4 important factors, i.e. study design, types of infections, outcome measures, and study locations. These subgroups analyses can help us to better assess the sources of variation or inconsistency of findings, and also better understand the specific subgroups of patients that may benefit more or less from statins. Thirdly, we assessed the quality of each study by well-established score scales (NOS and Jadad Score). The relatively high quality of most included studies can improve our interpretation of the pooled effect estimates for stains use.

### Study Limitations

Several limitations of this meta-analysis needed to be mentioned. First, we only included electronic database and published articles. Both Egger’s test and asymmetry of funnel plotpotential suggested the existence of publication bias. Second, this meta-analysis included more observational studies (n = 31) than clinical trials (n = 10). We did not assign different weighting to included studies based on the validity of their study design (RCT vs. observational studies). Third, 6 of the 10 included RCTs were designed to test the effect of statins on cardiac outcomes rather than infectious disease-related mortality. So the validity of estimated associations from these RCTs may be comprised. Fortunately, several ongoing clinical trials [60]–[63] (ID: NCT00528580, NCT00979121, NCT00702130, NCT00676897, http://www.clinicaltrials.gov) aim to specifically examine the potential clinical benefit of statins in sepsis. We expect these studies will yield more conclusive evidence on this important topic in near future.

### Conclusion

Based on this meta-analysis, we conclude that statins are associated with a lower risk of death in patients with infectious diseases in observational studies, but less in clinical trials. This beneficial effect tends to be short-term only. It seems to be stronger in patients with bacteremia but less for ICU patients with severe infection. More worldwide clinical trials specifically on this topic are urgently needed to provide more conclusive guideline for clinical practice.

## Supporting Information

Checklist S1 **PRISMA Checklist.**
(DOC)

Figure S1 **Forest plot of the association between statins and mortality (30-days) for patients with infectious disease.**
(TIF)

Figure S2 **Forest plot of the association between statins and mortality (90-days) for patients with infectious disease.**
(TIF)

Figure S3 **Forest plot of the association between statins and mortality (in-hospital) for patients with infectious disease.**
(TIF)

Figure S4 **Forest plot of the association between statins and mortality (long term) for patients with infectious disease.**
(TIF)

Figure S5 **Forest plot of the association between statins and mortality for patients with infectious disease, by study area.**
(TIF)

Flow Diagram S1 **PRISMA Flow Diagram.**
(DOC)
