# Supplementary figures and images for: Systematic Review and Meta-Analysis on the Association between Outpatient Statins Use and Infectious Disease-Related Mortality
Source: PLoS One. 2012 Dec 17;7(12):e51548. doi: 10.1371/journal.pone.0051548 (PMC3524177; doi:10.1371/journal.pone.0051548)

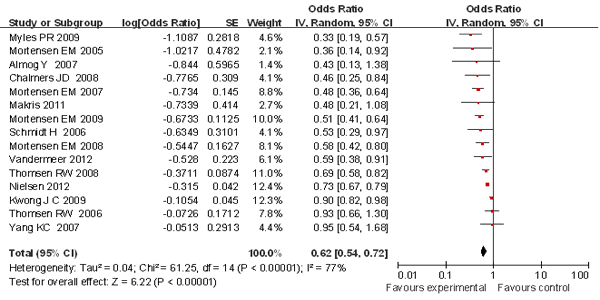

Supplement: Figure S1 — Forest plot of the association between statins and mortality (30-days) for patients with infectious disease. (TIF) [file pone.0051548.s002.tif]

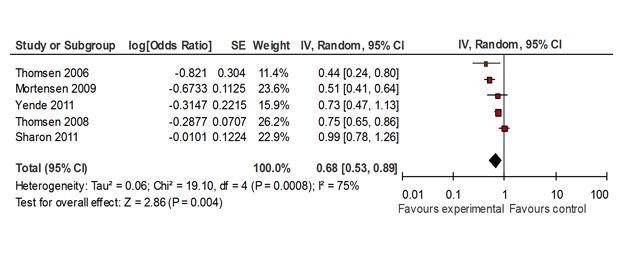

Supplement: Figure S2 — Forest plot of the association between statins and mortality (90-days) for patients with infectious disease. (TIF) [file pone.0051548.s003.tif]

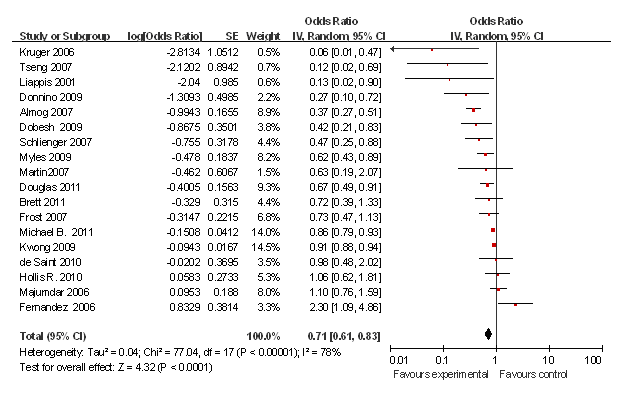

Supplement: Figure S3 — Forest plot of the association between statins and mortality (in-hospital) for patients with infectious disease. (TIF) [file pone.0051548.s004.tif]

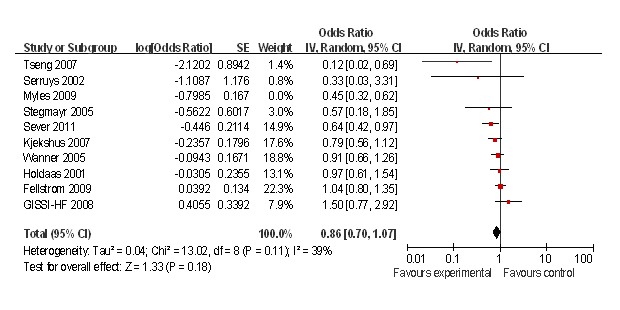

Supplement: Figure S4 — Forest plot of the association between statins and mortality (long term) for patients with infectious disease. (TIF) [file pone.0051548.s005.tif]

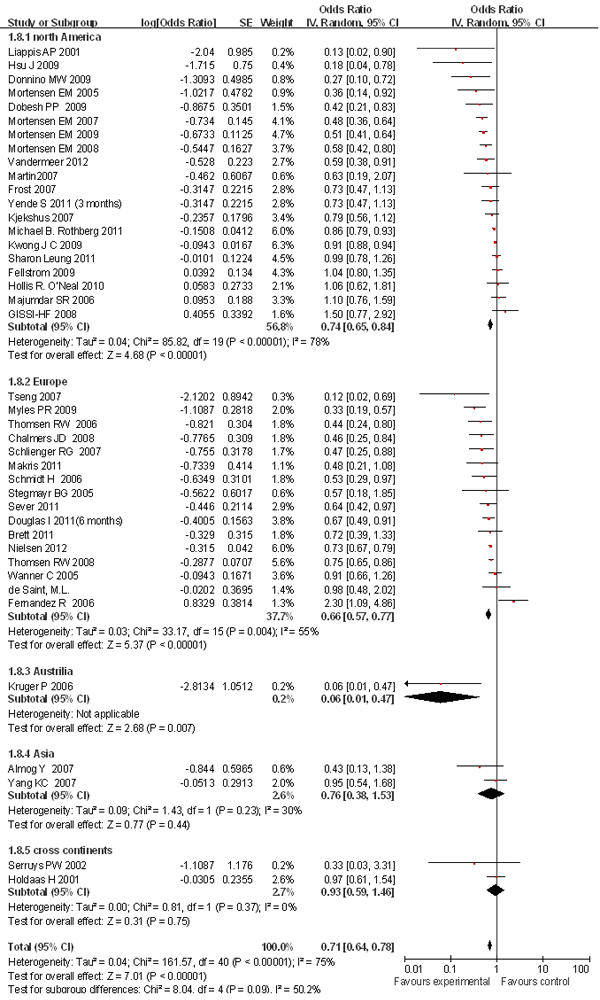

Supplement: Figure S5 — Forest plot of the association between statins and mortality for patients with infectious disease, by study area. (TIF) [file pone.0051548.s006.tif]
