# Supplementary material for: Systematic Review and Meta-Analysis on the Association between Outpatient Statins Use and Infectious Disease-Related Mortality
Source: PLoS One. 2012 Dec 17;7(12):e51548. doi: 10.1371/journal.pone.0051548 (PMC3524177; doi:10.1371/journal.pone.0051548)
Supplement: Flow Diagram S1 — PRISMA Flow Diagram. (DOC) [file pone.0051548.s007.doc]

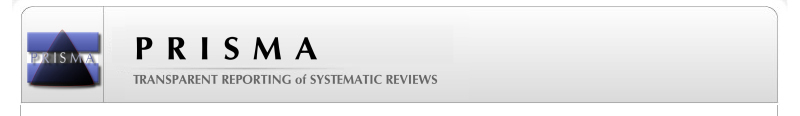
**PRISMA 2009 Flow Diagram**

**Screening**

**Included**

**Eligibility**

**Identification**

Records identified through database searching
(n =1458 )

Additional records identified through other sources
(n =0 )

Records after duplicates removed
(n =1453 )

Records screened
(n =406 )

Records excluded
(n = 240 )

Full-text articles assessed for eligibility
(n = 166 )

Full-text articles excluded, with reasons
(n = 125 )

Studies included in qualitative synthesis
(n = 41 )

Studies included in quantitative synthesis (meta-analysis)
(n =41 )
